# Comparison of the neuropsychological mechanisms of 2,6-diisopropylphenol and N-methyl-D-aspartate receptor antagonist against electroconvulsive therapy-induced learning and memory impairment in depressed rats

**DOI:** 10.3892/mmr.2015.3803

**Published:** 2015-05-21

**Authors:** GANG LIU, CHAO LIU, XUE NING-ZHANG

**Affiliations:** 1 Department of Anesthesiology, General Hospital of Beijing Military Area of PLA, Beijing 100700; 2 Department of Anesthesiology, Tianjin Chest Hospital, Tianjin 300222, P.R. China; 3 Department of Radiology, The Second Hospital of Tianjin Medical University, Tianjin 300211, P.R. China

**Keywords:** 2,6-diisopropylphenol, N-methyl-D-aspartate receptor antagonist, learning-memory abilities, electroconvulsive therapy, depression

## Abstract

The present study aimed to examine the neurophysiological mechanisms of the 2,6-diisopropylphenol and N-methyl-D-aspartate (NMDA) receptor antagonist against learning and memory impairment, induced by electroconvulsive therapy (ECT). A total of 48 adult depressed rats without olfactory bulbs were randomly divided into six experimental groups: i) saline; ii) 10 mg/kg MK-801; iii) 10 mg/kg MK-801 and a course of ECT; iv) 200 mg/kg 2,6-diisopropylphenol; v) 200 mg/kg 2,6-diisopropylphenol and a course of ECT; and vi) saline and a course of ECT. The learning and memory abilities of the rats were assessed using a Morris water maze 1 day after a course of ECT. The hippocampus was removed 1 day after assessment using the Morris water maze assessment. The content of glutamate in the hippocampus was detected using high-performance liquid chromatography. The expression levels of p-AT8^Ser202^ and GSK-3β^1H8^ in the hippocampus were determined using immunohistochemical staining and western blot analysis. The results demonstrated that the 2,6-diisopropylphenol NMDA receptor antagonist, MK-801 and ECT induced learning and memory impairment in the depressed rats. The glutamate content was significantly upregulated by ECT, reduced by 2,6-diisopropylphenol, and was unaffected by the NMDA receptor antagonist in the hippocampus of the depressed rats. Tau protein hyperphosphorylation in the hippocampus was upregulated by ECT, but was reduced by 2,6-diisopropylphenol and the MK-801 NMDA receptor antagonist. It was also demonstrated that 2,6-diisopropylphenol prevented learning and memory impairment and reduced the hyperphosphorylation of the Tau protein, which was induced by eECT. GSK-3β was found to be the key protein involved in this signaling pathway. The ECT reduced the learning and memory impairment, caused by hyperphosphorylation of the Tau protein, in the depressed rats by upregulating the glutamate content.

## Introduction

Depression is a common mental health concern (1) and electroconvulsive therapy (ECT) is the preferred treatment option for patients diagnosed with major depression. Electroconvulsive seizure for a duration of between 120 and 180 sec can lead to cognitive disorders (2) due to pathological dysfunction of the glutamic acid (Glu) signalling system (3–5). Glu is the predominant excitatory neurotransmitter, which transmits ~40% of synapses, causes oxidative stress (6) resulting in hippocampal indiscriminations and saturated long-term potentiation (LTP) (4), and causes synaptic plasticity impairment (5,7).

Tau proteins or τ proteins are low-molecular weight microtubule-associated proteins (8). These asymmetric phosphoproteins are distributed in the frontal and temporal lobes of the brain, axons and dendrons of the hippocampus and neurons in the entorhinal area. Their primary structures include the prominent feature of amino acid residue repeats in a 'tandem repeat' form, with the major Pro-Gly-Gly-Gly-structure at the carboxyl terminus. These repeats are involved in the microtubule-associated region of the Tau proteins, promoting the assembly and stabilisation of axon microtubules (9), maintaining the space between microtubules (10), affecting the axonal material transport of nerve cells, promoting neuronal growth and development, inhibiting lipid peroxidation and tubulin aggregation, and facilitating learning and memory (11). Tau protein phosphorylation is one of the predominant mechanisms regulating neuronal functions (12). The abnormal phosphorylation of Tau proteins induces misfolding and molecular aggregation (13), which consequently weakens their ability to stabilise microtubules and decrease axonal transfer efficiency, resulting in transmitter transport, storage and release disorders and synapse degeneration or changes in the distribution and activity of prion proteins (14). These phenomena can also cause neuronal apoptosis or death and lead to learning and memory impairment. Based on the presumed mechanism, Tau proteins can exert physiological functions as soluble DNA molecular chaperones. In addition, these proteins cannot bind with DNA during formaldehyde-induced degeneration or misfolding, causing a series of signal transduction impairments (15,16).

Tau protein hyperphosphorylation involves two mechanisms: Endogenous and exogenous. The former is triggered by the abnormal hyperphosphorylation of Tau protein in the brain, whereas the latter is triggered by dysequilibrium between protein kinases and phosphotases (17). GSK-3β is currently the Tau protein kinase with the most marked effect (18–20). It can catalyse Tau protein phosphorylation at multiple sites (21,22), including Thr^181^, Ser^199^, Ser^202^, Thr^205^, Thr^212^, Thr^217^, Thr^231^, Ser^396^ and Ser^404^. In addition, GSK-3β phosphorylates 9G8 and affects the alternative splicing of Tau exon 10, therefore, affecting the physiological function of the Tau proteins (23). Akt is at the core of the PIK/Akt signal transduction pathway (24) and is an upstream regulating factor of GSK-3β (25). Glu inhibits the activity of various protein kinases, including Akt (26).

The present study aimed to determine the effects of ECT at different electrical currents and for different durations on the hyperphosphorylation of Tau protein in depressed rats. The results may serve as a basis for further investigation of the molecular biological mechanisms underlying neural protection and clinical intervention therapy.

## Materials and methods

### Reagents and apparatus

The following reagents and apparatus were used throughout the present study: Pure 5-methyl-dihydro-propylcyclohepten-imine maleate (NMDA receptor antagonist or dizocilpine or MK-801 (Sigma-Aldrich, St. Louis, MO, USA), 2,6-diisopropylphenol (AstraZeneca Pharmaceuticals, Waltham, MA, USA), pure L-glutamic acid (Sigma-Aldrich), rabbit anti-human p-AT8^Ser202^ monoclonal antibody (GTX128164; GeneTex, Irvine, CA, USA), mouse anti-human GSK-3β^1H8^ monoclonal antibody (sc-377213; Santa Cruz Biotechnology, Inc., Santa Cruz, CA, USA), diaminobenzidine (DAB) colour development kit (Beyotime Institute of Biotechnology, Shanghai, China), Bicinchoninic acid (BCA) Protein Assay kit (Beyotime Institute of Biotechnology), o-phthalaldehyde (OPA) domestic analytical reagent (Beyotime Institute of Biotechnology), β-mercaptoethanol (Amresco LLC, Solon, OH, USA), chromatography-grade methanol (Beyotime Institute of Biotechnology), HPD-25D oil-free diaphragm vacuum pump (Sherali Seok Industrial Co., Ltd., Shanghai, China), YDJZ-II medical micro electric grinder (Huien Medical Instrument and Device Co., Ltd., Shanghai, China), Harvard sine-wave shock generator (NatureGene Corp., Medford, NJ, USA), Morris water maze video analysis system (Academy of Military Medical Sciences, Beijing, China), 5810R low-temperature high-speed centrifuge (Eppendorf, Hamburg, Germany), HPLC system (Waters, Milford, MA, USA), 18-ODS chromatography column (Dima Glass, Richmond Hill, ON, Canada), protein electrophoresis system (Bio-Rad Laboratories, Inc., Hercules, CA, USA), Goldisc multimedia image processing system (Chengdu Goldisc UESTC Multimedia Technology Co., Ltd., China) and Olympus-45 optical photomicrography system (Olympus, Tokyo, Japan).

### Animals

Healthy male Sprague-Dawley rats (24 weeks-old), weighing between 250 and 300 g were provided by the Department of Laboratory Animal Science, Tianjin Medical University (Tianjin, China). The rats were housed in a well-ventilated environment with free access to food and water, were subjected to an alternating 12 h light/dark cycle and were handled for 2 min daily to allow for acclimation.

Following 1 week of adaptive breeding, models of depressed rats without olfactory bulbs were established, as described previously (27,28). The rats were anaesthetised with an intraperitoneal injection of 2.75% sodium pentobarbital (55 mg/kg; Biyuntian Biotech Co., Ltd., Shanghai, China). The skin at the midpoint of the two ears was incised to expose the skull, and 2 mm intersections were cut between 7 and 8 mm in front of the anterior fontanels and on the two sides of the median raphes. Subsequently, two holes, 2 mm in diameter, were drilled using an electric grinder, and the olfactory bulb tissues were removed. The incisions were washed with penicillin solution (200,000 U/ml) and the skin was sutured. The rats were injected with 40,000 units penicillin sodium/day for three consecutive days.

The rats were handled and weighed each day following surgery, and the animals were subjected to open field assessment at 9 am for 2 weeks following recuperation from surgery. The open field assessment involved a box divided into 25 cells. The laboratory personnel placed the rats in the central square of the open field box. The rats were observed for 5 min to observe their movement, whereby 1 square was 1 point on the scoring system. The open field test mainly reflects the activity and the curiosity of the rats to novel environments therefore the open field test can evaluate the degree of depression in rats. A total of 48 rats with total horizontal and vertical scores ranging between 30 and 120 in the open field assessment were included in the experimental group.

### Principles for animal experimentation

The experiments in the present study were approved by the Hospital Ethics Committee of the First Affiliated Hospital of Chongqing Medical University (Chongqing, China). All animal experiments were performed according to the Principle for Treatment of Laboratory Animals issued by the American Medical Association and the Guide for the Care and Use of Laboratory Animals by the American Society of Animal Science and National Institutes of Health. All experiments and analyses were performed in a double-blinded manner, and the rats were raised in separate cages throughout the experiment.

### Grouping of laboratory animals

For the factorial design in analysis of variance (ANOVA), two intervention factors were included: ECT, comprising the ECT and without ECT groups; and drugs, comprising three groups (i.p injection of saline, 2,6-diisopropylphenol and MK-801).

### Intervention measures for laboratory animals

A total of 48 adult depressed rats without olfactory bulbs were randomly divided into six experimental groups (n=8 per group): I.p injection of 5 ml saline; ii) i.p injection of 5 ml of 10 mg/kg NMDA receptor antagonist MK-801 (29); iii) i.p injection of 5 ml 10 mg/kg MK-801 and a course of ECT; iv) i.p injection of 5 ml 200 mg/kg 2,6-diisopropylphenol (5); v) i.p injection of 5 ml 200 mg/kg 2,6-diisopropylphenol and a course of ECT; and vi) i.p injection of 5 ml saline and a course of ECT.

### ECT

Each group of corresponding drugs were administered by i.p injection 15 min prior to each ECT. The electrodes were placed at the bilateral temporal areas of the rats and a Harvard sine-wave ECT apparatus was used to provide electrical stimulation with a square wave (a single half sine wave of 20 ms) at a current of 50 mA and a frequency of 50 Hz for 1 sec. The occurrence of a tonic-clonic convulsion seizure was considered to indicate successful treatment (30). ECT was performed seven times at 9 am once every 2 days. The drugs were also injected into the rats in the experimental groups without ECT.

### Assessment of the learning and memory functions of experimental rats using a the Morris water maze video analysis system

The Morris water maze assessment was performed within 24 h after the rats had received ECT. The Morris water maze was equally divided into four quadrants: I, II, III and IV. Prior to training, the water maze was filled with tap water and ink was added to produce cloudy water, and a platform was placed 2 cm below the water surface in quadrant I. All experiments were performed between 9 and 3 pm in a quiet room with consistent article placement, lighting and a water temperature of 24±1°C. Morris 1.0 software (Academy of Military Medical Science) was used to track and record the data analyses. Place navigation assessments were performed on days 1–6. Briefly, the rats were placed in the water, facing the pool wall from quadrants I, II, III and IV in counterclockwise direction and observed for 120 sec. Prior to assessment, a platform was placed 2 cm under the water surface in the centre of quadrant I. The escape latency, which was the time during which the rats searched for and climbed the platform, was detected using a camera system (Olympus-45, Olympus, Tokyo, Japan). When the rats failed to find the platform within 120 sec, they were led back to the platform and the escape latency was recorded as 120 sec. Following assessment, the mean escape latency on days 1–6 was determined to indicate learning. The shorter the escape latency, the better the learning capacity of the rats. A space probe trial was performed on day 7. Briefly, the platform was removed and the rats were placed in the water facing the pool wall from quadrant III, furthest from the original platform. The duration of swimming of the rats in each quadrant within 60 was recorded using a camera system. The duration of swimming to the original platform in quadrant I (space probe duration) was used to determine the memory performance. The longer the duration, the better the memory capacity of the rats.

### Sample collection

As an important region closely associated with learning and memory functions in the brain, the hippocampus is involved in information acquisition, preservation and extraction, and is the predominant target area subject to injury by stress. Therefore, the hippocampus was selected as the region of investigation in the present study. The hippocampal tissues of the rats were extracted within 24 h after the Morris water maze assessment. The rats were fasted without water deprivation 8 h prior to sample collection and were subsequently anaesthetised by i.p injection of 20% ethyl carbamate (1.5 g/kg; Biyuntian Biotech Co., Ltd.). They were quickly sacrificed by decapitation to remove the brain tissues. The blood stains were soaked in ice-cold de-diethyl pyrocarbonate (Biyuntian Biotech Co., Ltd.) water to separate the bilateral hippocampal tissues. The left hippocampus was divided into sections A and B. Section A was frozen in liquid nitrogen overnight at −80°C in an ultra-low-temperature refrigerator for western blot analysis. Following weighing, section A was added to a 1 ml methanol-water centrifugate and subsequently homogenised at a low temperature. A sample of the homogenate was centrifuged at 10,000 × g at 4°C for 15 min and the supernatant was collected, filtered with a filter membrane and maintained at −80°C to measure the Glu content (*µ*g/g). The right hippocampus was fixed in 10% poly-formaldehyde (Biyuntian Biotech Co., Ltd.) at 4°C for 3 days, dehydrated, paraffin embedded, sectioned (thickness 1 *µ*m) and subsequently equipped for immunity organisation using the SP method.

### Determination of hippocampal Glu content of rats using HPLC (31)

The samples assessed were the treated supernatant of the hippocampal tissues of the rats. Reagents used in HPLC included Glu standard, OPA domestic analytical reagent (Beyotime Institute of Biotechnology), β-mercaptoethanol (Amresco LLC) and chromatography-grade methanol (Beyotime Institute of Biotechnology). Glu standard solutions at concentrations of 0.15, 0.30, 0.735, 1.47, 2.94, 3.675 and 5.88 mg/l were prepared and determined following derivatisation. The equipment used for chromotography included a 5810R low-temperature high-speed centrifuge, HPLC system equipped with a 600-series pump, a model 2475 fluorescence detector and an Empower chromatographic workstation and 18-ODS chromatographic column (Dima Glass) at 35°C. Mobile phase A contained 0.1 mol/l potassium acetate and mobile phase B contained methanol undergoing binary gradient elution. The mobile phase was filtered using a 0.45 *µ*m microporous filter membrane and subjected to ultrasonic degassing at a flow rate of 1.0 ml/min, excitation wavelength of 250 nm and emission wavelength of 410 nm. Quantification was determined based on the Glu peak area. For preparation of the derivatisation reagent, OPA (20 mg) was dissolved in 500 *µ*l methanol for ultrasonic dissolution, 500 *µ*l β-mercaptoethanol and 9 ml boric acid buffer solution (pH 10.0; Biyuntian Biotech Co., Ltd.) was added, and was subsequently stored between 0°C and 4°C. For preparation of the amino acid standard solution, standard solution (100 *µ*mol/l) was prepared using the Glu standard and was diluted prior to assessment.

### Derivatisation and analysis

Either the standard solution (100 *µ*l) or tissue sample solution was placed in an microcentrifuge tube and subsequently reacted with 100 *µ*l derivatisation reagent for 2 min, with 20 *µ*l of sample injected at 20°C.

### Establishment of the Glu standard curve

Glu standard solutions at concentrations of 0.15, 0.30, 0.735, 1.47, 2.94, 3.675 and 5.88 mg/l were prepared and determined following derivatisation. Quantitative analysis was performed using the external standard method, and the concentrations (X) and peak areas (Y) were subjected to linear regression to obtain a linear equation.

### Determination of hippocampal Glu content

The homogenate supernatant from the hippocampal tissue was thawed and added to 2 ml frozen formic acid (1 mol/l; Biyuntian Biotech Co., Ltd.), following which the mixture was homogenised manually in an ice bath. The homogenate was centrifuged at 8×10^12^ × g for 30 min at 4°C and the supernatant was maintained at −20°C for subsequent use. The homogenate supernatant (1 ml) of the brain tissue was added to 0.75 ml 4% sodium bicarbonate solution (Biyuntian Biotech Co., Ltd.) and subsequently centrifuged at 3.5×10^12^ × g for 5 min at 4°C. The supernatant was filtered using a 0.45 *µ*m filter membrane and then loaded. Subsequently, 24 *µ*l loaded solution was collected and 12 *µ*l derivatisation reagent and 960 *µ*l sodium tetraborate buffer solution (pH 9.18; Biyuntian Biotech Co., Ltd.) was added to the sample and allowed to stand at 20°C for 3 min. Sampling and gradient elution were then performed to determine the Glu content. The sampling was performed using the sampling tube of the HPLC chromatographic system and the gradient elution was performed using an 18 ODS chromatographic column (Dima Glass).

### Assessment of the protein expression levels of p-AT8^Ser202^ and GSK-3β^1H8^ in the hippocampus of rats using the SP method

The parrafin embedded hippocampal tissues were dewaxed, hydrated with ethanol at 20°C, flushed with distilled water and subsequently immersed in 0.01 mol/l phosphate-buffered saline (PBS; Biyuntian Biotech Co., Ltd.) for 5 min, 3% H_2_O_2_ for 15 min at 20°C and 0.01 M citric acid buffer (Biyuntian Biotech Co., Ltd., Shanghai, China) with a liquid hydrogen ion index of 6.0 for 15 min. The samples were subsequently boiled for 15–20 min for antigen repair, cooled for 20 min at room temperature and incubated in 10% sheep serum albumin (Biyuntian Biotech Co., Ltd., Shanghai, China) for 15 min at 20°C. The regular SP method was performed as follows: 50 *µ*l antibodies, including rabbit anti-human p-AT8^Ser202^ monoclonal antibody and mouse anti-human GSK-3β^1H8^ monoclonal antibody, at a dilution of 1:400, at 37°C for 2 h. Subsequently, 50 *µ*l immunoglobulin G and 50 *µ*l S-A (horseradish peroxidase) were added to each slide. A 3,3′-diaminobenzidine (DAB) colour reaction was then performed. For the negative control, the primary antibodies were replaced with 0.01 mol/l PBS. The number of positive cells under each high power field (10 rats/group, 10 slides/rat, 10 high power fields/slide) were determined under a light microscope (BM-E biological microscope; Leica Microsystems GmbH, Wetzlar, Germany) and the average optical density value of the positive cells was measured using a multimedia image handling system.

### Determination of the protein expression levels of p-AT8^Ser202^ and GSK-3β^1H8^ in the rat hippocampus using western blot analysis

The hippocampal tissues of the rats were homogenised, and 0.2 g homogenate was placed in cell lysis buffer (Biyuntian Biotech Co., Ltd., Shanghai, China) for western blot analysis, and immunoprecipitation buffer (Biyuntian Biotech Co., Ltd., Shanghai, China) to extract proteins. The protein concentration was determined using a BCA protein assay kit and adjusted for consistency. Equal quantities (30 *µ*g) of the extracted protein samples were diluted with 5X sodium dodecyl sulphate (SDS) sample loading buffer solution (Biyuntian Biotech Co., Ltd., Shanghai, China) at a ratio of 1:1 (v/v) and boiled at 100°C for 5 min. The mixture of the pre-stained protein molecular weight markers was further dissolved in 1X SDS sample loading buffer solution and then boiled at 100°C for 3 min. The samples (15 *µ*l aliquot of each) were loaded, using glyceraldehydes-3-phosphate dehydrogenase for calibration, and subjected to SDS-polyacrylamide gel electrophoresis (PAGE) until the target molecular weight was achieved. The protein bands were electrically transferred to polyvinylidine fluoride membranes (Bio-Rad Laboratories, Inc.) using a wet transfer process. The membrane was then blocked with 50 g/l non-fat milk powder for 3 h and subsequently incubated with rabbit anti-human p-AT8^Ser202^ monoclonal antibody (1:400) and mouse anti-human GSK-3β^1H8^ monoclonal antibody (1:400) at 4°C overnight. The immunoglobulin G (1:200) was marked using the corresponding horseradish peroxidase and then incubated at 37°C for 2 h. A DAB kit was used for colour development and a Goldisc multimedia image processing system was used to determine the integral absorbance value of the positive bands.

### Statistical analysis

The data are expressed as the mean ± standard deviation. Homogeneity of variances for each sample group were assessed using SPSS 19.0 statistical software (SPSS, Inc., Chicago, IL, USA). Each group was subjected to factorial design and one-way analysis of variance (ANOVA) to determine the predominant effects and interaction effects of each treatment factor. The effect of each treatment factor was analysed using one-way ANOVA, and multiple comparisons were determined using the least significant difference test and Student Newman-Kuels-q-test. P<0.05 was considered to indicate a statistically significant difference.

## Results

### Detection of learning and memory functions of the rats using the Morris water maze video analysis system: Escape latency and space probe time

The ECT and drug (2,6-diisopropylphenol and NMDA receptor antagonist) treatments resulted in learning and memory impairment in the rats, with prolonged escape latency (ECT, *F*=148.986 and P<0.001; NMDA receptor antagonist and 2,6-diisopropylphenol, *F*=3.809 and P=0.030) and a shortened space probe time (ECT, *F*=4.376 and P=0.043; NMDA receptor antagonist and 2,6-diisopropylphenol, *F*=17.863 and P<0.001). However, these effects presented a negative association (escape latency, *F*=32.870 and P<0.001; space probe time, *F*=98.938 and P<0.001). The combination of ECT and Glu receptor antagonist or 2,6-diisopropylphenol alleviated learning and memory impairment in rats (Tables I and II).

### Detection of Glu content in rat hippocampus using HPLC

ECT (*F*=277.841 and P<0.001) and 2,6-diisopropylphenol (*F*=21.320 and P<0.001) significantly increased the concentration of Glu in the hippocampus, and these effects were negatively associated. The NMDA receptor antagonist had no significant effect on the concentration of Glu in the hippocampus (Table III).

### Determination of the protein expression levels of p-AT8^Ser202^ and GSK-3β^1H8^ in the rat hippocampus using the SP method

**Figure 1:**
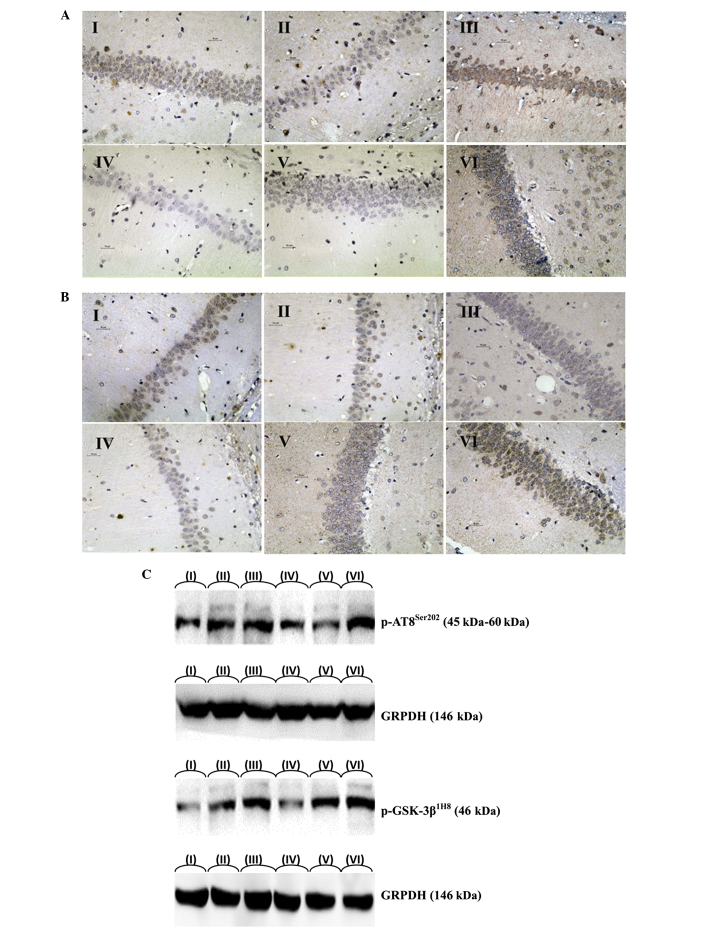
Representative images of the expression levels of (A) p-AT8^Ser202^ and (B) GSK-3β^1H8^ in the hippocampus of rats (magnification, ×400; n=8). (C) Protein expression levels of p-AT8^Ser202^ and GSK-3β^1H8^ in the hippocampal tissues of depressed rats without olfactory bulbs. I. i.p injection of 5 ml saline; II, i.p injection of 5 ml 10 mg/kg NMDA receptor antagonist (MK-801); III, i.p injection of 5 ml 10 mg/kg NMDA receptor antagonist (MK-801) and a course of ECT; IV, i.p injection of 5 ml 200 mg/kg 2,6-diisopropylphenol; V, i.p injection of 5 ml 200 mg/kg 2,6-diisopropylphenol and a course of ECT; VI, i.p injection of 5 ml saline and a course of ECT. ECT, electroconvulsive therapy.

ECT increased the protein expression levels of p-AT8^Ser202^ and GSK-3β^1H8^ in the hippocampus of rats, as indicated by the quantity of IR-positive cells (p-AT8^Ser202^, *F*=255.037 and P<0.001; GSK-3β^1H8^, *F*=98.216 and P<0.000) and the integral absorbance value of the IR-positive cells (p-AT8^Ser202^, *F*=366.698 and P<0.001; GSK-3β^1H8^, *F*=167.764 and P<0.001). However, the NMDA receptor antagonist and 2,6-diisopropylphenol reduced the expression levels of p-AT8^Ser202^ and GSK-3β^1H8^ in the hippocampus of the rats, as indicated by the quantity of IR-positive cells (p-AT8^Ser202^, *F*=56.003 and P<0.001; GSK-3β^1H8^, *F*=71.848 and P<0.001) and the integral absorbance value of the IR-positive cells (p-AT8^Ser202^, *F*=56.003 and P<0.001; GSK-3β^1H8^, *F*=71.848 and P<0.001). These effects presented a negative association. The NMDA receptor antagonist and 2,6-diisopropylphenol slowed the ECT-induced increase of the protein expression of Tau in the hippocampus of the rats (Fig. 1A and B; Tables II–IV), as indicated by the quantity of IR-positive cells (p-AT8^Ser202^, *F*=3.507 and P=0.039; GSK-3β^1H8^, *F*=3.651 and P=0.035) and the integral absorbance value of the IR-positive cells (p-AT8^Ser202^, *F*=40.174 and P<0.001; GSK-3β^1H8^, *F*=11.247 and P<0.001). Therefore, the NMDA receptor antagonist and 2,6-diisopropylphenol slowed the ECT-induced increase in protein expression of phosphorylated Tau in the hippocampus, (Fig. 1A and B; Tables IV–VII).

### Determination of the protein expression levels of p-AT8^Ser202^ and GSK-3β^1H8^ in the rat hippocampus using western blot analysis

ECT increased the protein expression levels of p-AT8^Ser202^ and GSK-3β^1H^ (p-AT8^Ser202^, *F*=350.725 and P<0.001; GSK-3β^1H8^, *F*=35.412 and P<0.001), whereas 2,6-diisopropylphenol and the NMDA receptor antagonist reduced their expression levels (p-AT8^Ser202^, *F*=73.129 and P<0.001; GSK-3β^1H8^, *F*=68.465 and P<0.001). These effects presented a negative association (p-AT8^Ser202^, *F*=4.580 and P=0.016; GSK-3β^1H8^, *F*=5.698 and P=0.006). For example, 2,6-diisopropylphenol and the NMDA receptor antagonist slowed the ECT-induced increase in the protein expression of Tau, and the key regulatory protein may be GSK-3β^1H8^ (Fig. 1C; Tables VIII and IX).

## Discussion

### Glu and learning-memory impairment

In the present study, significant post-ECT increased hippocampal Glu concentration was accompanied with decreased learning and memory abilities, as indicated by prolonged escape latency and shortened space probe duration, the former indicating impaired learning ability and the latter indicating a decline in explicit memory. These findings were in agreement with those of previous studies (3–5), in which ECT was demonstrated to induce Glu-associated excitotoxicity. In addition, this process was revealed to be proportional to the current and duration of ECT, which further confirmed the observation. The NMDA antagonist alleviated the spatial learning and memory impairment induced by the overexcitation of GluR, which was consistent with the experimental result of Wu *et al* (32) that post-ECT decline in learning and memory abilities are a result of oxidative stress, caused by the overexcitation of GluR, and result in hippocampal LTP saturation and synaptic plasticity impairment.

The hippocampus of the limbic system is important in memory. The episodic memory in the explicit memory depends on the hippocampus (33). The hippocampus is not only closely associated with short-term memory, but also with long-term spatial memory in rats (34). The water maze assessment was used to determine the spatial memory in the episodic memory. The spatial memory of humans or animals is summarised in the cognitive map stored in the hippocampus (35). Hippocampal cells can receive and process spatial information from different sources, enabling cognitive map formation or increased synaptic contact of cell assemblies in the association cortex to form the permanent memory of spatial positions (36). The present study demonstrated that increased hippocampal Glu concentration and the increased hyperphosphorylation of the Tau protein caused impairment of the spatial memory of the rats. By contrast, 2,6-diisopropylphenol partially inhibited the excitotoxicity of Glu and further alleviated the hyperphosphorylation of the Tau protein. These results also confirmed that the hippocampal tissues function in the spatial memory and explicit memory of rats.

Palmio *et al* (37) indicated that ECT was unable to impair neurons, as the post-ECT neuron-specific enolase and S-100B proteins in the serum were not significantly increased. These findings are inconsistent with those of the present study. Whether this result is associated with the limited sample size (10 individuals) requires further confirmation.

In the present study, 2,6-diisopropylphenol decreased the post-ECT hippocampal Glu content and improved the post-ECT learning and memory abilities of the rats. This finding was consistent with those of previous studies (38). However, the NMDA receptor antagonist caused learning and memory impairment and partially alleviated post-ECT learning and memory impairment. The NMDA receptor antagonist revealed no significant effect on hippocampal Glu content. Therefore, the NMDA receptor antagonist may function by inhibiting the excitability of Glu instead of decreasing the excretion of Glu in the hippocampus. Additionally, the excitability of the NMDA receptor at normal levels is essential for learning and memory, which is also consistent with a previous study (39).

In comparative analysis of the present study with previous reports, in contrast to the present study, Stover *et al* (40) revealed that 2,6-diisopropylphenol increases the concentration of Glu in the cerebrospinal fluid. This finding may be attributed to the patients undergoing neurosurgery and the effect of the interference factor (neurosurgery) on the concentration of Glu in the nervous system, which is higher compared with the anesthesia treatment. Previous studies also demonstrated that 2,6-diisopropylphenol failed to protect the Glu content of rat cortex and hippocampal tissues from injury (41). At high doses, 2,6-diisopropylphenol can aggravate injury and even increase the release of Glu (42). This finding is in disagreement with that of the present study, and the *in vitro* brain slices used may not be appropriate to simulate the *in vivo* environment. In contrast to the present study, Pesić *et al* (43) revealed that 2,6-diisopropylphenol induces cortical neuron death. This inconsistency in results may be attributed to the fact that the rats used in this previous study were 7-day-old neonates, therefore, their nervous system was immature and more sensitive to drugs, compared with adult rats.

Learning and memory abilities are associated with the hyperphosphorylation of Tau protein, in that learning and memory abilities decrease as the phosphorylation of Tau protein is upregulated (16). In the present study, increased levels of phosphorylated hippocampal Tau proteins prolonged the escape latency and shortened the space probe duration of the rats, revealing impaired learning ability and explicit memory, respectively. Therefore, Tau proteins may exert physiological effects as soluble DNA molecular chaperones, which cannot bind with DNA during formaldehyde induced degeneration or misfolding (44).

Phosphorylation at the pAT8^Ser202^ site was also observed to be closely associated with ECT intervention, consistent with the results of previous studies (23,45,46). However, the explanation of its signal transduction pathway and action site differed. Jeon *et al* (47) indicated that the process is performed by serine/threonine protein kinase-1 at the Ser^262^ site. However, the present study demonstrated that the Ser^202^ site and ECT-induced Tau hyperphosphorylation may be more important.

The present study demonstrated that the expression levels of all phosphorylated hippocampal Tau proteins were upregulated as the post-ECT Glu concentration in the neurons increased, and decreased axonal transport efficiency caused further accumulation of Glu in the brain. However, Tau proteins cannot bind with DNA and their function as molecular chaperones was affected, resulting in neuronal dysfunction. This result is in agreement with the results of Wu *et al* (32), who revealed that stress can activate the excitatory neurotransmission system to induce hippocampal Tau protein hyperphosphorylation. The results of the present study are also consistent with those reported by Tan *et al* (48), who demonstrated that low body temperature, rather than 2,6-diisopropylphenol, can induce the Tau protein hyperphosphorylation.

Vossel *et al* (49) demonstrated that NMDA receptor excitation can activate the polymerisation of GSK-3β, casein kinase 2 and actin to increase the phosphorylation of Tau, which impairs neurons. By contrast, NMDA receptor antagonists inhibit the excitotoxicity of Glu at the receptor level to interrupt nerve injury caused by GSK-3β-induced Tau protein hyperphosphorylation (50,51).

The present study demonstrated that the increased hippocampal Glu concentration was accompanied by a decline in learning and memory. However, the learning and memory impairment caused by the overexcitation of GluR was alleviated following treatment with the GluR antagonist. This finding was similar to that of previous studies (38).

The present study demonstrated that the ECT-induced increase of hippocampal Glu concentration and increased hippocampal Tau protein hyperphosphorylation were a dose- and time-dependent. Among all the hyperphosphorylation sites, phosphorylation at the pAT8^Ser202^ site was the most closely associated with ECT stress, and may also have been involved in learning and memory impairment caused by Tau protein hyperphosphorylation. This result may be associated with Tau protein phosphorylation at the Ser^202^ site, positioned in the microtubule binding domain, being involved in regulating the binding activity of Tau proteins and microtubules.

Following ECT, GSK-3β^1H8^, a key protein which regulates Tau protein phosphorylation, was also upregulated, together with p-AT8^Ser202^, and the upregulated expression levels of GSK-3β^1H8^ and p-AT8^Ser202^ were reversed following intervention of 2,6-diisopropylphenol and NMDA receptor antagonist. This result is consistent with that of Muyllaert *et al* (52), which demonstrated that GSK-3β activity is regulated by serine and tyrosine phosphorylation, indicating that the relevant signal transduction pathways of 2,6-diisopropylphenol and the NMDA receptor antagonist are affected by the key protein, GSK-3β^1H8^.

Therefore, the present study hypothesized the following signal transduction pathways: ECT induces increases in hippocampal Glu concentration, whereas Glu excites the ionotropic receptor (GluR), inhibits the Akt signal channel, increases the expression and activity of GSK-3β, and increasesthe phosphorylation of Tau protein in the hippocampus, thereby decreasing axonal transport efficiency, and promoting neural signal transmission impairment and synaptic degeneration, causing neuronal apoptosis or death. Hippocampal Tau protein hyperphosphorylation can induce neurotransmitter transport impairment, further resulting in the accumulation of Glu in the injured neuron, forming cycles that aggravate neuronal injury.

Previous studies indicated that 2,6-diisopropylphenol can reduce the Glu content of the brain and inhibit neuronal apoptosis by improving the activity of Akt (53). In the present study, 2,6-diisopropylphenol lowered the post-ECT hippocampal concentration of Glu, which was consistent with a study by Xu *et al* (54). In addition, 2,6-diisopropylphenol decreased Tau protein hyperphosphorylation to improve post-ECT learning and memory abilities, therefore it was suggested that 2,6-diisopropylphenol affected the signaling pathway of the enhancement of ECT-induced Tau protein phosphorylation in two aspects: 2,6-diisopropylphenol reduced the Glu concentration of the brain and improved Akt activity, the latter possibly being a secondary effect of the former.

2,6-diisopropylphenol also reduced the expression of GSK-3β^1H8^, decreasing hippocampal Tau protein hyperphosphorylation and improving post-ECT spatial learning and memory abilities. This result is similar to the findings of Straiko *et al* (55), who revealed that ketamine and 2,6-diisopropylphenol inhibits protein kinase phosphorylation through the same pathway as that of lithium in inhibiting enzyme phosphorylation.

Vossel *et al* (49) demonstrated that NMDA receptor excitation activates the polymerisation of GSK-3β, casein kinase 2 and actin to increase the phosphorylation of Tau, thereby injuring neurons. However, the NMDA receptor antagonist can inhibit the excitotoxicity of Glu at the receptor level (50,51,56–62), interrupting the neuronal injury caused by GSK-3β induced Tau protein hyperphosphorylation. In the present study, the NMDA receptor antagonist decreased the expression of GSK-3β^1H8^, therefore, decreasing hippocampal Tau protein hyperphosphorylation and improving post-ECT spatial learning and memory abilities. This result is consistent with those of previous studies (35,50,51,56–60). However, there are different views on the specific site of Tau protein phosphorylation, which has the closest association with excitation by the NMDA receptor (61–63).

Kingston *et al* (64) revealed that 2,6-diisopropylphenol can inhibit the phosphorylation of NMDA receptor NR1 subunits through the signal transduction pathway of serine/threonine phosphatase PP2A, thereby decreasing the activity of the NMDA receptor (45). This conclusion further demonstrated that 2,6-diisopropylphenol functions directly as an NMDA receptor antagonist (65) and partially reverses post-ECT learning and memory impairment (66).

The results of the present and previous studies demonstrated that 2,6-diisopropylphenol functions directly as an NMDA receptor antagonist and partially reverses post-ECT learning and memory impairment (65). ECT, as a marked inducer of stress, induces increased hippocampal Glu concentration, while Glu excites the ionotropic receptor GluR, therefore, inhibiting the Akt signaling pathway (67), decreasing the activity of Akt, weakening the inhibition of GSK-3β by Akt (68), increasing the activity of GSK-3β, increasing hippocampal protein Tau phosphorylation, decreasing axonal transport efficiency, impairing neural signal transmission and causing neuron apoptosis or death, leading to the learning and memory impairment. Tau hyperphosphorylation resulted in neurotransmitter transport impairment and further induced Glu accumulation in injured neurons, forming cycles and aggravating neuron injury. GluR and GSK-3β were key nodes in this mechanism. It has been suggested that hippocampal Tau protein hyperphosphorylation induced by ECT is slowed, and space learning and memory impairment is alleviated correspondingly if one link is inhibited, therefore, the use of 2,6-diisopropylphenol or NMDA receptor antagonist to reduce Glu in brain and 2,6-diisopropylphenol can be used as an NMDA receptor antagonist.

The present study demonstrated that the stress of ECT induced increased hippocampal Glu concentration and Tau protein phosphorylation and decreased learning and memory abilities, and was positively associated with the current and duration of ECT. Glu excites the NMDA receptor and AMPAR to increase hippocampal Tau protein phosphorylation, affecting the alternative splicing of Tau exon 10 and decreasing axonal transport efficiency, resulting in neural signal transmission impairment and synaptic degeneration, finally leading to learning and memory impairment (69). In addition, hippocampal Tau protein hyperphosphorylation can result in neurotransmitter transport impairment, causing further accumulation of Glu in the injured neurons, forming cycles that aggravate neuronal injury (70).

As demonstrated in the present study, inhibition of the 2,6-diisopropylphenol or NMDA receptor antagonist links, slowed the ECT induced hippocampal Tau protein phosphorylation and alleviated spatial learning and memory impairment. Furthermore, the NMDA receptor was as important as the AMPA receptor for GluR in the above process, indicating that the role of AMPA in the neuromolecular biological mechanism of learning and memory is similar to that of the NMDA receptor.

The present study demonstrated that GSK-3β was a key protein in the signaling pathway regulating Tau phosphorylation. ECT-induced increase of hippocampal Glu concentration increased the hippocampal Tau protein phosphorylation, leading to learning and memory impairment. Glu excites the iontropic receptors, NMDA and AMPA, to inhibit the Akt signaling pathway, thereby decreasing the activity of Akt activity, weakening GSK-3β inhibition by Akt, increasing GSK-3β activity, increasing hippocampal Tau protein phosphorylation and decreasing axonal transport efficiency. This series of events resulted in neural signal transmission impairment, synaptic degeneration and neuronal apoptosis or death, leading to learning and memory impairment. Hippocampal Tau protein hyperphosphorylation in the hippocampus resulted in neurotransmitter transport impairment, further causing accumulation of Glu in the injured neurons, forming cycles that aggravate neuronal injury. GluR, Akt and GSK-3β are key factors in this signal transduction pathway and, as demonstrated in the present study, inhibiting 2,6-diisopropylphenol slowed ECT induced hippocampal Tau protein phosphorylation and alleviated spatial learning and memory impairment.

The findings indicate that increased apoptosis may be an explanation for the reduced OB volume and olfactory dysfunction in patients with depression. In addition, the mitochondrial-dependent death pathway may be involved in apoptosis in the OB of the rats.

## Figures and Tables

**Table I:** Morris water maze assessment of escape latency.

Group	Saline	NMDA receptor antagonist	2,6-diisopropylphenol	Total	F-statistic	P-value
Control	26.65±3.39	36.35±3.30	34.59±3.91	32.53±5.48	17.009	0.000
ECT	61.68±8.26	45.77±5.53	43.93±4.34	50.46±10.10	19.425	0.000
Total	44.17±19.09	41.06±6.56	39.26±6.26	41.49±12.11	3.809^a^	0.030^a^
F-statistic	123.107	17.102	20.463	148.986^a^	–	–
P-value	0.000	0.001	0.000	0.000^a^	–	–

Data are expressed as the mean ± standard deviation (n=8). Crossover effect, F=32.870 and P<0.001;

a F-statistic and P-value of main effect; ECT, electroconvulsive therapy.

**Table II:** Morris water maze assessment of space probe duration.

Group	Saline	NMDA receptor antagonist	2,6-diisopropylphenol	Total	F-statistic	P-value
Control	26.36±4.21	11.17±1.69	11.86±1.51	16.46±7.62	77.237	0.000
ECT	10.92±2.30	16.38±2.16	17.67±1.65	14.99±3.58	24.363	0.000
Total	18.64±8.61	13.78±3.28	14.76±3.37	15.73±5.94	17.863^a^	0.000^a^
F-statistic	82.814	28.928	54.291	4.376^a^	–	–
P-value	0.000	0.000	0.000	0.043^a^	–	–

Data are expressed as the mean ± standard deviation (n=8). Crossover effect, F=98.938 and P<0.001;

a F-statistic and P-value of main effect; ECT, electroconvulsive therapy.

**Table III:** Glu content in the rat hippocampus (*µ*mol/gprot).

Group	Saline	NMDA receptor antagonist	2,6-diisopropylphenol	Total	F-statistic	P-value
Control	46.51±9.35	49.43±9.77	36.90±6.25	44.28±9.87	4.652	0.021
ECT	162.16±31.89	149.93±24.86	92.32±16.34	134.80±39.32	17.553	0.000
Total	104.34±63.89	70.88±25.69	64.61±31.02	89.54±53.82	21.320^a^	0.000^a^
F-statistic	96.903	113.283	80.278	277.841^a^	–	–
P-value	0.000	0.000	0.000	0.000^a^	–	–

Data are expressed as the mean ± standard deviation (n=8). Crossover effect, F=11.091 and P<0.001;

a F-statistic and P-value of main effect; ECT, electroconvulsive therapy.

**Table IV:** Number of p-AT8^Ser202^ IR-positive cells the in rat hippocampus.

Group	Saline (n)	NMDA receptor antagonist (n)	2,6-diisopropylphenol (n)	Total (n)	F-statistic	P-value
Control	39.13±6.94	21.50±3.59	20.25±2.76	26.96±9.92	38.964	0.000
ECT	80.13±11.63	48.88±6.77	53.13±8.48	60.71±16.64	27.257	0.000
Total	59.63±23.11	35.19±15.07	36.69±18.04	43.83±21.78	56.003^a^	0.000^a^
F-statistic	73.329	102.138	108.781	255.037^a^	–	–
P-value	0.000	0.000	0.000	0.000^a^	–	–

Data are expressed as the mean ± standard deviation (n=8). Crossover effect, F=3.507 and P=0.039;

a F-statistic and P-value of main effect; ECT, electroconvulsive therapy.

**Table V:** Integral absorbance value of positive cells in rat hippocampus (p-AT8^Ser202^-IR).

Group	Saline	MDA receptor antagonist	2,6-diisopropylphenol	Total	F-statistic	P-value
Control	0.1807±0.0135	0.0727±0.0119	0.0644±0.0114	0.1060±0.0554	222.375	0.000
ECT	0.4095±0.0521	0.1585±0.0200	0.1634±0.0125	0.2438±0.1238	150.951	0.000
Total	0.2951±0.1238	0.1156±0.0471	0.1139±0.0524	0.1749±0.1177	279.136^a^	0.000^a^
F-statistic	144.283	108.566	273.455	366.698^a^	–	–
P-value	0.000	0.000	0.000	0.000^a^	–	–

Data are expressed as the mean ± standard deviation (n=8). Crossover effect, F=40.174 and P<0.001;

a F-statistic and P-value of main effect; ECT, electroconvulsive therapy.

**Table VI:** Quantity of positive cells in rat hippocampus (GSK-3β^1H8^-IR).

Group	Saline	NMDA receptor antagonist	2,6-diisopropylphenol	Total	F-statistic	P-value
Control	35.88±7.04	15.25±3.06	14.38±2.97	21.83±11.12	52.452	0.000
ECT	65.25±13.47	32.75±6.06	31.13±6.62	43.04±18.37	33.947	0.000
Total	50.56±18.38	24.00±10.16	22.75±9.97	32.44±18.45	71.848^a^	0.000^a^
F-statistic	29.895	53.096	42.607	98.216^a^	–	–
P-value	0.000	0.000	0.000	0.000^a^	–	–

Data are expressed as the mean ± standard deviation (n=8). Crossover effect, F=3.651 and P=0.035;

a F-statistic and P-value of main effect; ECT, electroconvulsive therapy.

**Table VII:** Integral absorbance value of positive cells in rat hippocampus (GSK-3β^1H8^-IR).

Group	Saline	NMDA receptor antagonist	2,6-diisopropylphenol	Total	F-statistic	P-value
Control	0.1012±0.1169	0.0645±0.0043	0.0634±0.0063	0.0815±0.0267	106.291	0.000
ECT	0.1673±0.0132	0.0873±0.0072	0.0899±0.0062	0.1148±0.0389	187.156	0.000
Total	0.1421±0.0288	0.0758±0.0132	0.0767±0.0150	0.0982±0.0371	291.269^a^	0.000^a^
F-statistic	62.186	60.189	71.389	167.764^a^	–	–
P-value	0.000	0.000	0.000	0.000^a^	–	–

Data are expressed as the mean ± standard deviation (n=8). Crossover effect, F=11.247 and P<0.001;

a F-statistic and P-value of main effect; ECT, electroconvulsive therapy.

**Table VIII:** Western blot analysis and integral absorbance value of p-AT8^Ser199/202^ protein content in rat hippocampus.

Group	Saline	NMDA receptor antagonist	2,6-diisopropylphenol	Total	F-statistic	P-value
Control	695.38±151.65	405.25±74.39	399.88±89.92	500.17±176.16	23.879	0.000
ECT	1354.38±94.42	913.13±76.87	888.13±71.15	1051.88±232.17	50.175	0.000
Total	1024.88±361.52	659.19±272.26	644.00±264.02	776.02±345.37	73.129^a^	0.000^a^
F-statistic	119.457	101.646	149.425	350.725^a^	–	–
P-value	0.000	0.000	0.000	0.000^a^	–	–

Data are expressed as the mean ± standard deviation (n=8). Crossover effect, F=4.580 and P=0.016;

a F-statistic and P-value of main effect; ECT, electroconvulsive therapy.

**Table IX:** Western blot analysis and integral absorbance value of GSK-3β^1H8^ protein content in rat hippocampus.

Group	Saline	NMDA receptor antagonist	2,6-diisopropylphenol	Total	F-statistic	P-value
Control	496.75±98.35	327.25±62.21	329.75±52.06	384.58±107.29	13.937	0.000
ECT	700.75±52.42	400.13±61.09	392.75±58.00	497.88±156.46	75.294	0.000
Total	598.75±129.98	363.69±70.45	361.25±62.39	441.23±144.53	68.465^a^	0.000^a^
F-statistic	26.806	5.589	5.227	35.412^a^	–	–
P-value	0.000	0.033	0.038	0.000^a^	–	–

Data are expressed as the mean ± standard deviation (n=8). Crossover effect, F=5.698 and P=0.006;

a F-statistic and P-value of main effect; ECT, electroconvulsive therapy.
